# One Small Step for Rhinos, One Giant Leap for Wildlife Management- Imaging Diagnosis of Bone Pathology in Distal Limb

**DOI:** 10.1371/journal.pone.0068493

**Published:** 2013-07-09

**Authors:** Gabriela Galateanu, Thomas B. Hildebrandt, Alexis Maillot, Pascal Etienne, Romain Potier, Baptiste Mulot, Joseph Saragusty, Robert Hermes

**Affiliations:** 1 Department of Reproduction Management, Leibniz Institute for Zoo and Wildlife Research, Berlin, Germany; 2 Parc zoologique d’Amnéville, Amnéville, France; 3 Parc zoologique de La Barben (Pélissane), La Barben, France; 4 ZooParc de Beauval, Saint-Aignan, France; INRA, France

## Abstract

Chronic foot disease poses a threat to the general health, represents a tremendous clinical challenge, and often is a reason for euthanasia in captive megaherbivores, among them the elephant and rhinoceros. Nevertheless, apart from the elephant, foot pathology is handled as being confined only to soft tissues whereas bone pathology is often overlooked. As a case in point, the authors selected the second largest mammal on land, the rhinoceros. We performed a computed tomographic (CT) study using the highest resolution available in veterinary world, followed by digital radiography of eight distal limbs from two white and one Indian rhinoceroses. Our study demonstrated that bone pathology in rhinoceroses’ foot is present and in large numbers, yet none of these were diagnosed ante mortem. Even when the animals were euthanized due to foot problems, the decision was based on soft tissue pathology rather than orthopedic reasons. Even more worrying is the fact that the largest number of osteopathologies was present in one of the white rhinoceroses that showed no discernable related clinical signs. This study describes for the first time the existence of bone pathology in white rhinoceros foot, in addition to the two previously described rhinoceros species - Indian and black rhinoceroses. Furthermore, the chronic foot disease reported for the Indian rhinoceros in our study was not restricted to soft tissue structures as was presumed ante mortem but included severe bone pathology. New evidence suggesting that osteopathology in rhinoceroses’ distal limb is more widespread than it was thought before could force us to rethink of radiographic diagnosis in captive megaherbivores as routine examination incorporated into their health management. The anticipated improvements in radiologic examinations in megaherbivores will increase the effectiveness of their management and husbandry and open the way for improved animal welfare and better wildlife conservation.

## Introduction

Except for the elephant [1], [2], other wild animals in captivity are rarely trained to be handled and managed. Therefore, any diagnostic procedure should be performed following sedation or, more commonly, general anaesthesia, a procedure not without its own risks [3]–[5]. Due to difficulties in approaching non-domestic animals, many diagnostic procedures are simply not done, overlooked or performed too late. Additionally, as a protection mechanism, wild animals are known to disguise any sign of disease or clinical symptoms until late stages when they cannot be concealed any longer [6], [7]. The combination of these two hindrances results in under-diagnosis of various disorders in wild animals under captive conditions and the consequential delayed treatment.

One case in point is the chronic foot disease in captive herbivores which is frequently reported, yet, the radiologic diagnosis is performed in very few isolated situations [8]. As a result, foot pathology is often regarded as if it were restricted to soft tissue only, whereas bone lesions are being overlooked. Without accurate diagnosis and, consequently, proper medical treatment, foot diseases in captive wildlife pose threat to their general health and welfare, impacts on breeding ability, represents a tremendous clinical challenge and often result in fatal consequences, being a reason for euthanasia [9]–[11].

This situation is best demonstrated in foot disorders, which are a major health concern in both captive elephants and rhinoceroses [12]–[15]. Captive elephants display a wide variety of both soft tissue and bone pathologies of the distal limb. Foot osteopathology of captive elephants comprises a large variety of lesions including angular limb deformity, arthritis (supurative and chronic), degenerative joint disease (DJD), delayed physeal closure, dislocations, exostoses, fractures, osteomyelitis, and osteophytosis [15]–[18].

Unlike in elephants, the reported rhinoceroses’ foot pathologies have long been known for being confined to soft tissues, such as chronic pododermatitis in Indian rhinoceros [11], [13], [19], while documented skeletal afflictions are very scarce. Osteoarthritis in one black rhinoceros [20] and osteomyelitis in one Indian rhinoceros [21] and one Eastern black rhinoceros [22] are the only reported skeletal pathologies in the rhinoceros’ foot. Since bone pathologies in all mammals have universal etiologies, it is not clear why so little has been reported in rhinoceroses.

This discrepancy grabbed our attention and, in order to clarify it, we deliberately selected the second largest mammal on land after the elephant [12], [23], the rhinoceros. The authors initiated a high-resolution computed tomographic (CT) study followed by digital radiography (DR). Bone pathologies of the rhinoceros distal limb have only very rarely been reported, but the present study shows that these are highly prevalent and diverse.

## Materials and Methods

### Rhinoceroses

Eight distal limbs (four front legs and four hind legs) from three captive rhinoceroses, obtained post mortem, were used for this study. Distal limb encompassed the autopodium and its related soft-tissue structures represented by the hand (manus) or foot (pes), being composed of podial elements (carpus/tarsus), metapodials (metacarpus/metatarsus) and phalanges [24].

#### Rhinoceros 1

Left distal hind limb excerpt was obtained from a 38-year-old male white rhinoceros (*Ceratotherium simum*). This individual presented a medial, large tumefaction on its hind left limb diagnosed histologically as epidermoid carcinoma.

#### Rhinoceros 2

Three distal limbs (two front and left hind) were collected from a 38-year-old male white rhinoceros with no reported foot disease.

#### Rhinoceros 3

All four distal limbs of a 24-year-old female Greater Asian one-horned, or Indian, rhinoceros (*Rhinoceros unicornis*) were obtained following euthanasia. Ante- mortem the animal suffered from chronic foot infection in all four limbs for many years.

Rhinoceroses 1 and 3 were euthanized due to foot related diseases and rhinoceros 2 was euthanized due to other, unrelated pathologies.

The legs of the two white rhinoceroses were sectioned above the carpal and tarsal joints (included), while the Indian rhinoceros’ legs were sectioned at the level of carpal and, respectively, tarsal joints (partially included). Therefore, the total number of bones included in this study was 203 instead of 220.

### Computed Tomographic Data Acquisition and Imaging

Computed tomographic data was acquired from all eight distal limbs using a third-generation, 128-slice scanner (Aquilion CX, Toshiba Medical Systems Cooperation, Tochigi, Japan). Settings for the CT helical scan protocol were: 120 kV, 100–300 mA, 0.6 s rotation time and 0.5 mm acquisition slice thickness. The reconstruction protocol included both body-standard and bone-high resolution algorithms with a reconstruction slice thickness/slice interval of both 1/0.8 mm and 0.5/0.25 mm.

ViTREA® 2 version 4.0 medical imaging software (Vital Images Inc., Minnetonka, MN, USA) provided the tools for two-dimensional (2D) and tri-dimensional (3D) analysis of CT images. We used volume-rendering software, simultaneous imaging of specific anatomical and pathological structures of interest using a combination of 2D orthogonal Multi-Planar Reconstructions (MPR) and 3D images; trim function with 3D and 2D segmentation to focus images on regions of interest, a wide variety of clinical viewing protocols, and fine adjustment of visualization parameters to enhance the diagnostic quality of the images. Oblique and curved MPRs were necessary in order to delineate several fractures with a complex 3D architecture.

### Digital Radiography

Digital radiography was conducted on all seven distal limbs from rhinoceros 2 and rhinoceros 3, using a mobile x-ray unit (Mobi X-Ray, SEDECAL, Madrid, Spain) with exposure parameters of 100–250 mA, 250 kVp, and exposure time of 0.050–0.100 s. For each foot, eight radiographic views were performed. Fifty-six digital radiographs were assessed for depiction of bone pathology. The standardized nomenclature for radiographic projections in veterinary medicine was used [25], [26]. The projections performed were four orthogonal projections: dorso-palmar (plantar) [DPa(l)], palmaro (plantaro)-dorsal [Pa(l)D], medio-lateral [ML], latero-medial [LM], and four oblique projections: dorsomedial-palmaro (plantaro) lateral [DM-Pa(l)LO], dorsolateral-palmaro (plantaro) medial [DL-Pa(l)MO], palmaro (plantaro) medial-dorsolateral [Pa(l)M-DLO] and palmaro (plantaro) lateral-dorsomedial [Pa(l)L-DMO].

### Statistical Analysis

Statistical analysis was performed using PASW Statistics 18 (formerly SPSS, IBM Inc., Chicago, IL). The Chi-square goodness-of-fit exact test was used to test whether the observed proportions for categorical variables differ from the hypothesized equal distribution.

Rhinoceros 1 suffered from epidermoid carcinoma on the only limb available from this animal. As this tumor may have been the cause for at least some of the osteopathologies found in this foot and thus may have biased the statistical analysis, we have also analyzed our data after excluding this animal. Results indicate no biasing effect of rhinoceros 1 as none of the comparisons changed in a way that alter our findings (data not shown). Results are therefore shown for all three rhinoceroses combined.

A *P*-value <0.05 was considered statistically significant for all statistical tests.

## Results

**Figure 1:**
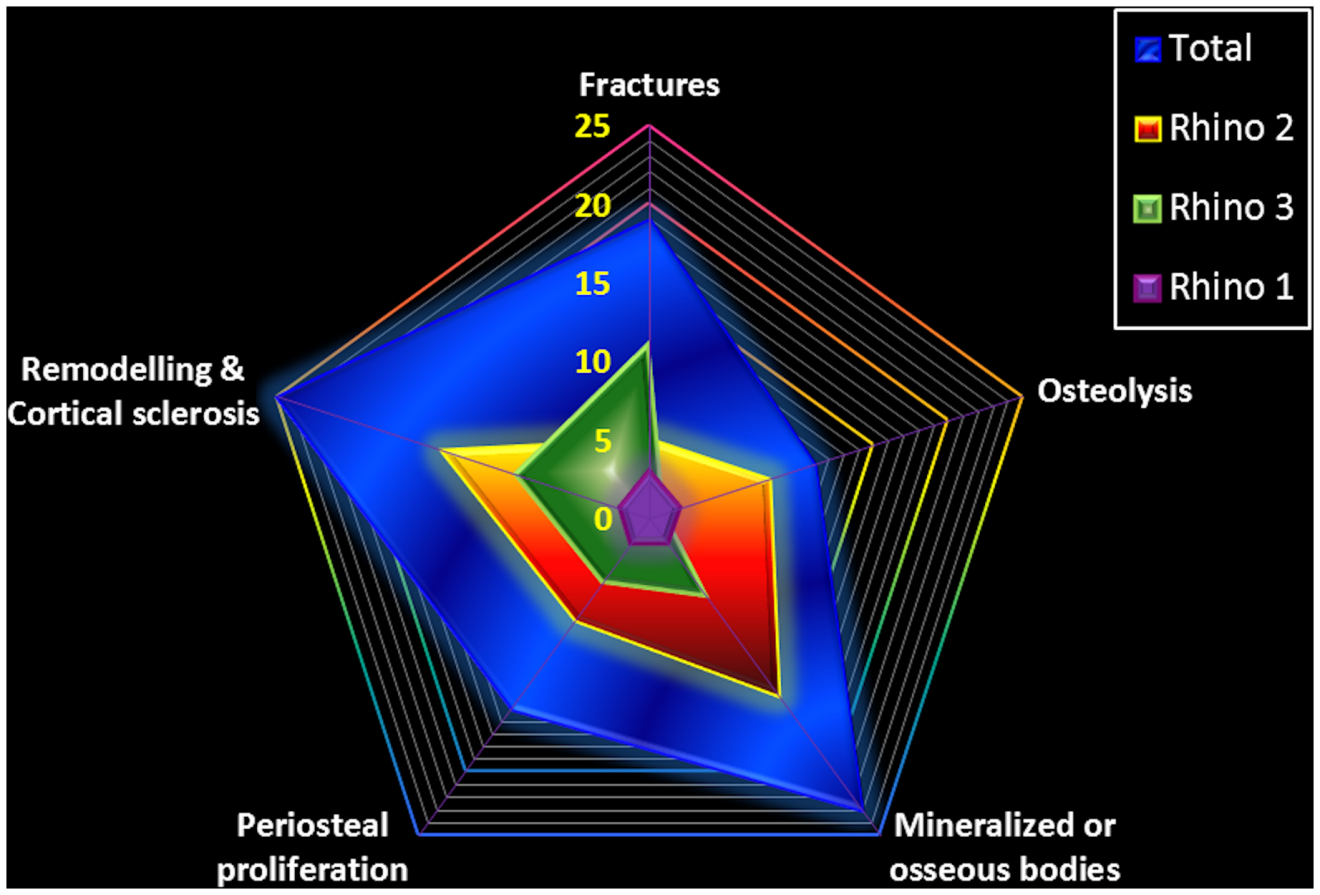
Main types of bone pathologies and their distribution in studied rhinoceroses. Chart representing the most frequent osteopathologies encountered in each rhinoceros and all three together.

**Figure 2:**
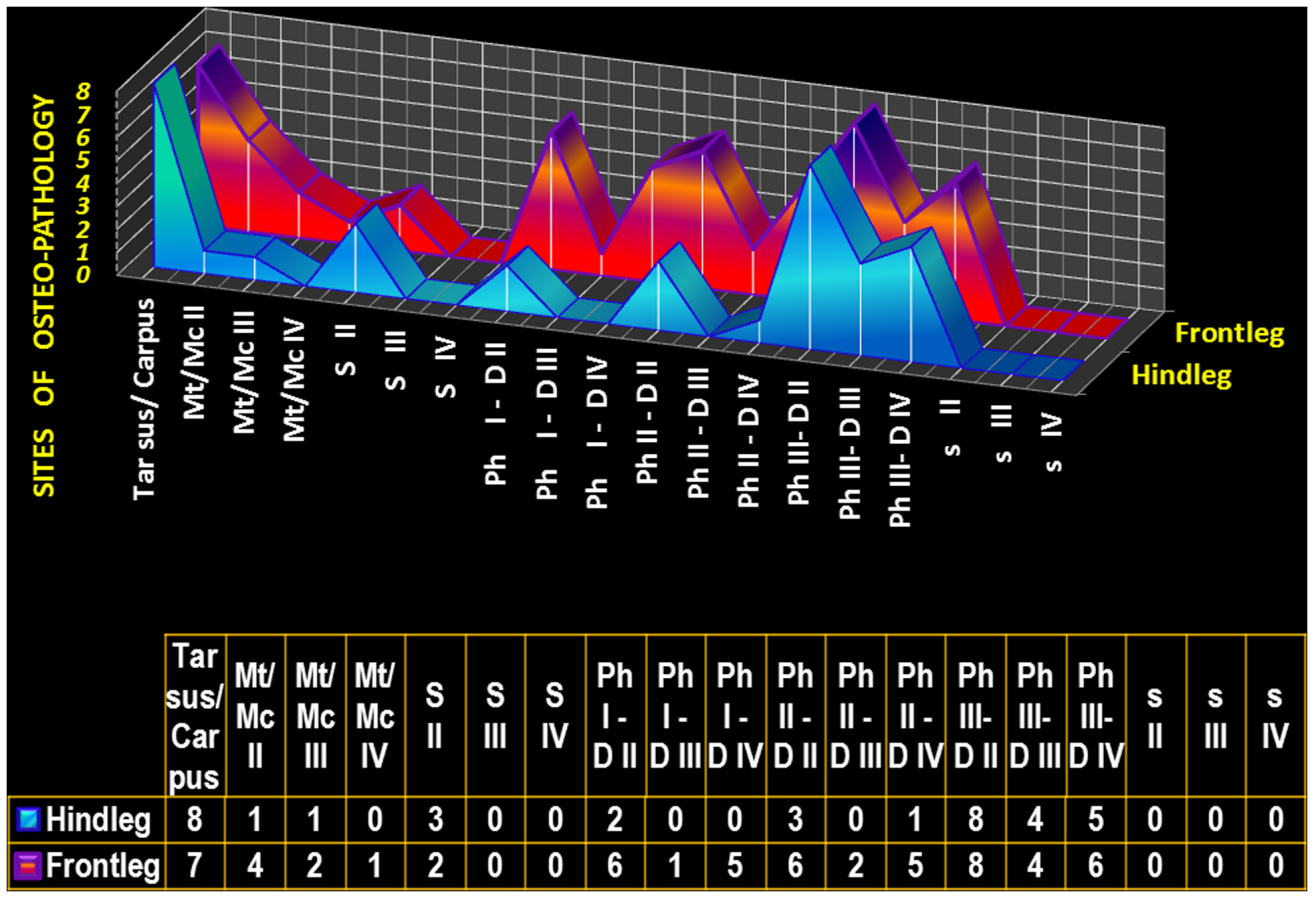
Distribution of bone pathologies in rhinoceroses’ autopodium. Graphic numerical representation of the pathological bone sites (vertical axis) found in each autopodial element (horizontal axis). For podial elements (carpal and tarsal bones), the number of osteopathologies per bone was small and therefore we included the carpal (n = 7) and, respectively, tarsal (n = 8) joint as one unit. The data was collected from all four hind legs (“Hindleg”) and from all four front legs (“Frontleg”), therefore, for example, “Ph I-DII” of “Hindleg” represents the first phalanx of the second digit in all four hind legs. Abbreviations: Mt-metatarsal, Mc-metacarpal, S-large sesamoids, Ph-phalanx, D-digit, s-small sesamoids.

**Figure 3:**
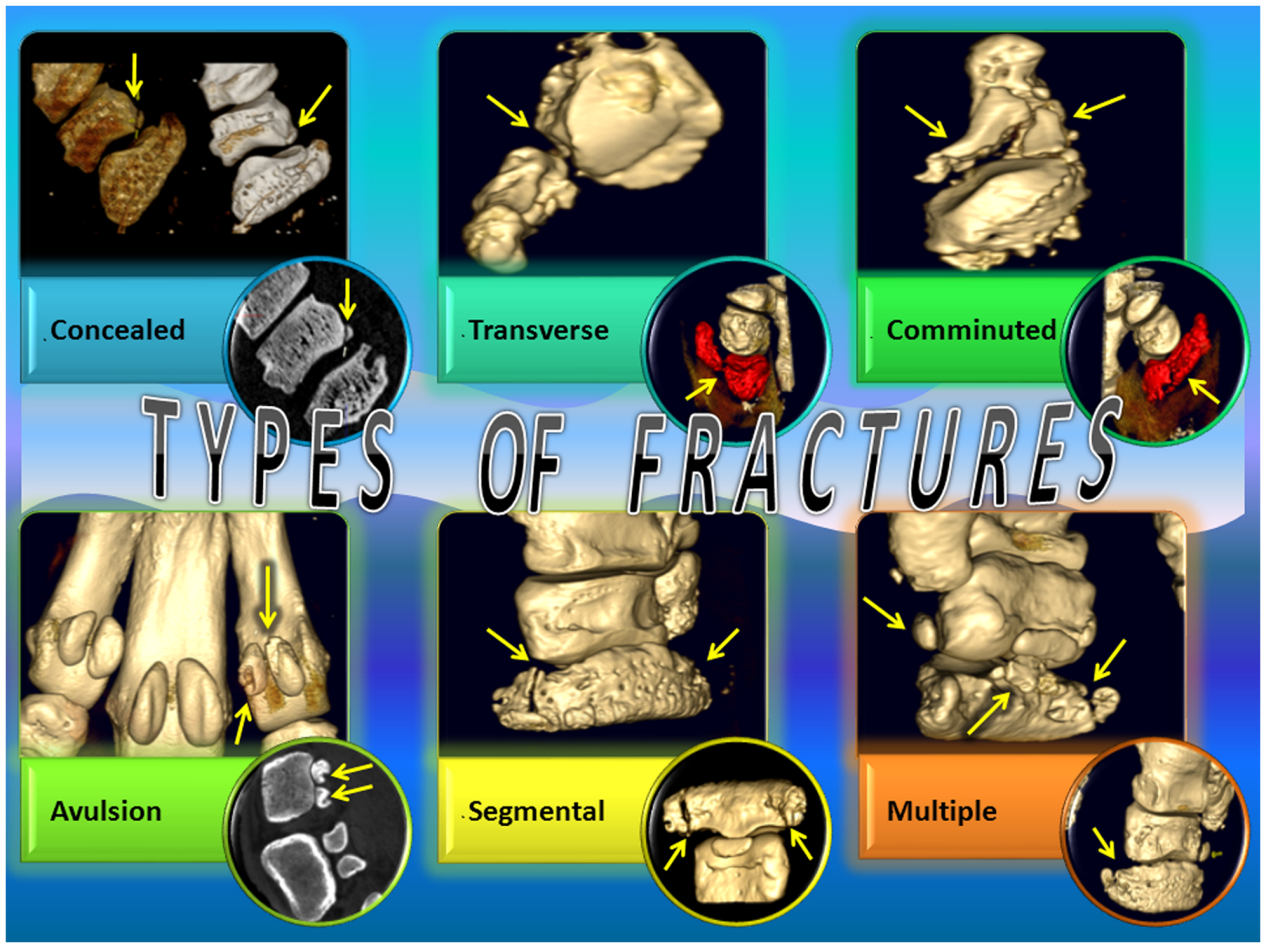
Types of fractures. Computed tomographic images of different types of fractures: *concealed fractures*, characterized by new bone production overlying the fracture line, concealing it; *transverse fractures*, complete, occurring perpendicular on the long axis of the bone; *comminuted fractures*, with minimum three bone fragments and connected fracture lines; *avulsion fractures*, separation of small fragments of bone due to traction by soft tissue attachment; *segmental fractures*, many separate fracture lines in a single bone; *multiple fractures*, fractures affecting different bones.

The prevalence of the main bone pathologies in each rhinoceros is shown in Fig. 1. The number of bone lesions for each autopodial element is shown in Fig. 2 and the main findings in each of the three rhinoceroses are shown in Table 1. Computed tomographic images of different types of fractures are shown in Fig. 3.

**Table 1:** Bone pathologies in three rhinoceroses.

	Rhinoceros	WR 1	WR 2			IR			
	Limb	HL	FR	FL	HL	FR	FL	HR	HL
**Osteolysis**	**Focal**	CTB		DII:Mc,PhI-III					
	**Diffuse**	TI	DII:Mc,S,PhI-III; DIV:PhI		DII:PhIII DIV:PhIII				DIII:PhIII
	**Bone cysts**				DII:PhII				
	**Fractures**	CTB; DIV:PhIII	DIV:PhI	U; DII:S	DII:S,PhII,PhIII; DIV:PhIII	DII:PhIII; DIV:PhIII	DIII:PhI-III; DIV:PhII-III		DII:PhIII; DIII:PhIII
**Osteogenesis**	**Periosteal reaction**	MtII; MtIII	DII:Mc,S,PhI-III	DII:PhI-III		DIV:PhII			DII:PhI-III; DIV:PhIII
	**Cortical sclerosis**	CTB	DII:Mc,S,PhI-III						DII:PhI-II; DIV:PhIII
	**Bone remodelling**	DII:S	DII:Mc,S,PhI-III; DIV:S,PhI- II	U; I; R; DII:S,PhI-III		DIV:PhI- II	DIV:PhII-III	DI-III:PhIII	DII:PhIII
	**Mineralised bodies**	DII:PhIII; DIV:PhIII	CIII; DII:PhIII; DIV:PhIII	R; DII:PhI-III	Ca; Ta; DII:PhIII; DIV:PhIII	DIV:Mc,PhI,PhII	DIV:Mc,PhII	DIII:PhIII	
	**Ankylosis**	CTB-TI							

WR 1- white rhinoceros 1, WR 2- white rhinoceros 2; IR- Indian rhinoceros, FR-front right, FL-front left, HR-hind right, HL-hind left autopodium, R-radial carpal bone, I-intermediate carpal bone, U-ulnar carpal bone, Ta-talus, Ca-Calcaneus, CTB-central tarsal bone, T I-first tarsal bone, Mc-metacarpal bone, Mt-metatarsal bone, D-digit, Ph-phalanx, S-large or proximal sesamoids. The lesions visible on digital radiographs are underlined.

**Figure 4:**
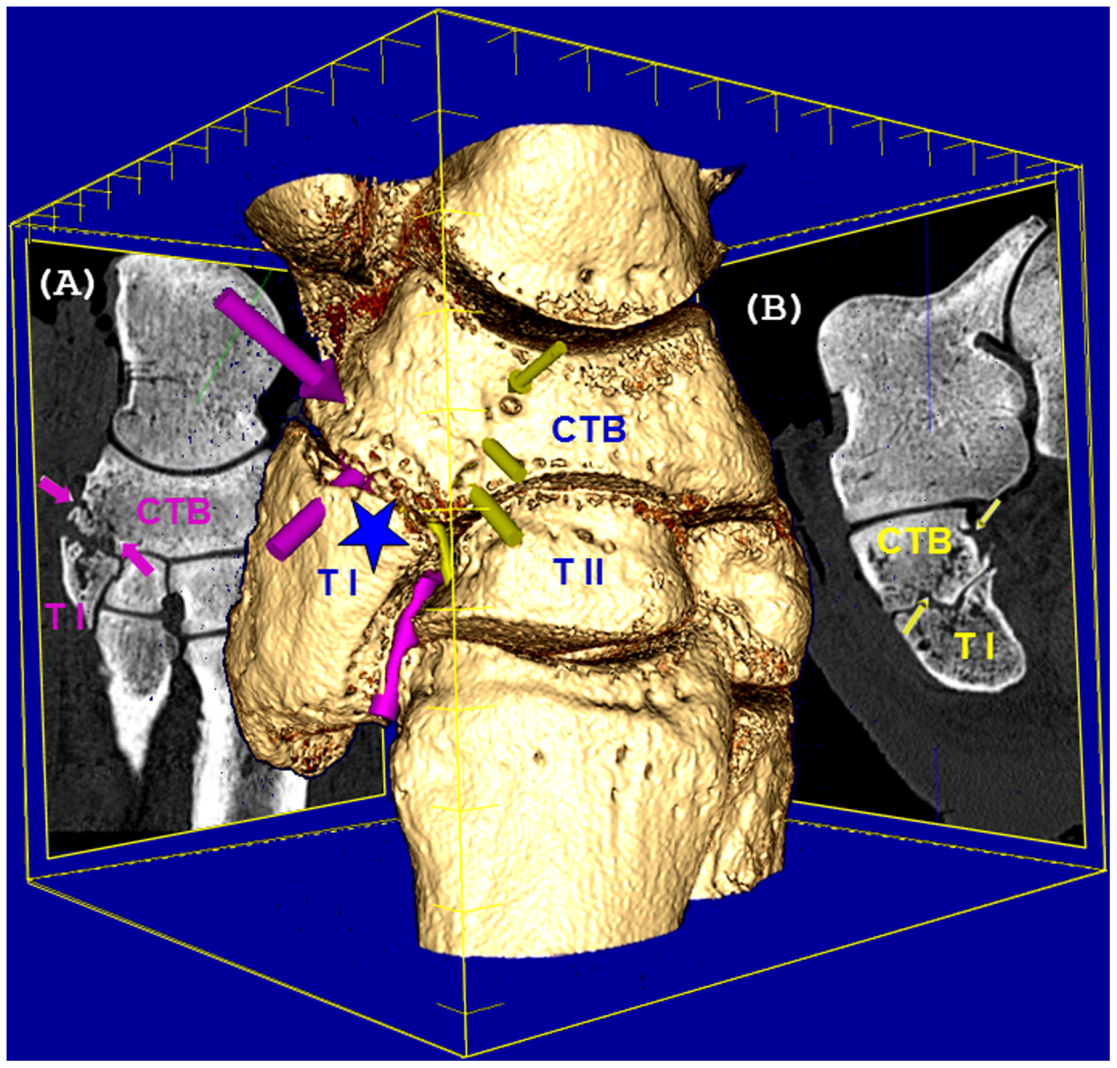
Left central tarsal bone (CTB) fractures in rhinoceros 1. Tridimensional computed tomographic reconstruction of the left tarsal joint with oblique multiplanar reconstructions (MPR) showing minimally displaced, multiple fractures of the left CTB situated in different planes (arrows). First line of fracture (A) descends in proximo-plantar to distalo-dorsal direction (large arrows), ending at the junction between CTB, first tarsal bone (T I) and second tarsal bone (T II). The second line of fracture (B) is oriented from dorsal to plantar surfaces, in proximo-medial to distalo-plantar direction, reaching the midline of the proximal articular surface of T I with CTB (small arrows). At the level of these fractures, CTB distalo-medial aspect reveals a mixed pattern of trabecular focal bone loss (osteolysis) and cortical osteogenesis represented by massive, unstructured new bone production and remodelling, with a beak-like formation oriented plantaro-medially, hook-shaped in axial plane. Additionally, the articular surface between CTB and TI is highly irregular, characterized by decreased joint space width, articular bone proliferation that bridges the contiguous bones (ankylosis), erosion and lysis of the articular cartilage and underlying bone (asterisk).

**Figure 5:**
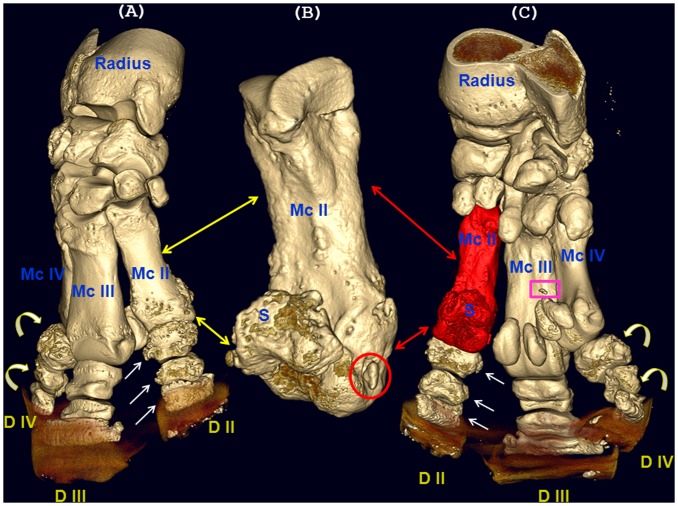
Bone pathology of the distal right front limb in rhinoceros 2. Computed tomographic tridimensional (A–C) images: (A) autopodium, medio-dorsal aspect; (B) second metacarpal bone, latero-palmar aspect; (C) autopodium, latero-palmar aspect. Second metacarpal bone (Mc II) exhibits amorphous periosteal reaction adjunct to severe remodelling of the distal epiphysis and metaphysis, associated with underlying cortical lysis prolonged in a proximal direction to the mid-diaphysis, where the cortex is irregularly thinned (long, double headed arrows). Cortical bone proliferation, periosteal reaction, and severe remodelling are also present on the neighbouring sesamoidal (S) bones (fused, with bridging bony spurs) and phalanges (Ph) I, II, III of the second digit (D II), (simple and double headed short arrows). The second phalanx and the paired sesamoids of the fourth digit (D IV) express moderate cortical remodelling, in contrast with the first phalanx where the cortical lysis (mainly dorsal) and bone remodelling are extensive, accompanied by multiple, bony fragments (curved arrows). Mineralized body (C-rectangle) and bone fragments (B-circle) are evident.

**Figure 6:**
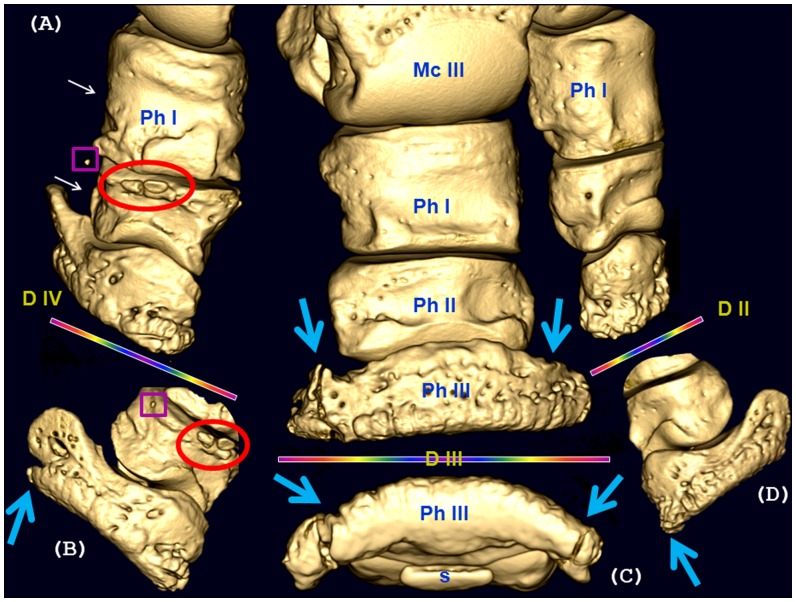
Bone pathology of the distal right front limb in rhinoceros 3. Dorsal aspect of the distal autopodium (A) and close-up computed tomographic images of distal phalanges (Ph) from lateral (D IV) digit (B), middle (D III) digit (C) and medial (D II) digit (D) showed multiple osteopathologies. Distal phalanges (Ph III) of each digit present fractures (large arrows) characterized as follows: medial digit (D II) – complete, with dorso-distal triangular fractured fragment of bone; central digit (D III) - severe, comminuted fracture of the lateral palmar process, with two main fragments (20×30×10 mm and 10×20×10 mm) accompanied by callus formation and also severe fracture of the medial palmar process with one fragment; lateral digit (D IV) - chip fracture of the distal part of lateral palmar process. Other pathologic changes observed on D IV include: small mineralized body (2 mm diameter, A, B-rectangle) between the first phalanx (Ph I) and the second phalanx (Ph II); two medium sized osseous bodies (10 mm length, A, B-circle) situated dorso-proximally to Ph II; dorso-medial amorphous periosteal reaction on the proximal half of Ph II; moderate bone remodelling of Ph I and Ph II (small arrows). The small sesamoid (s) of D III is intact.

**Figure 7:**
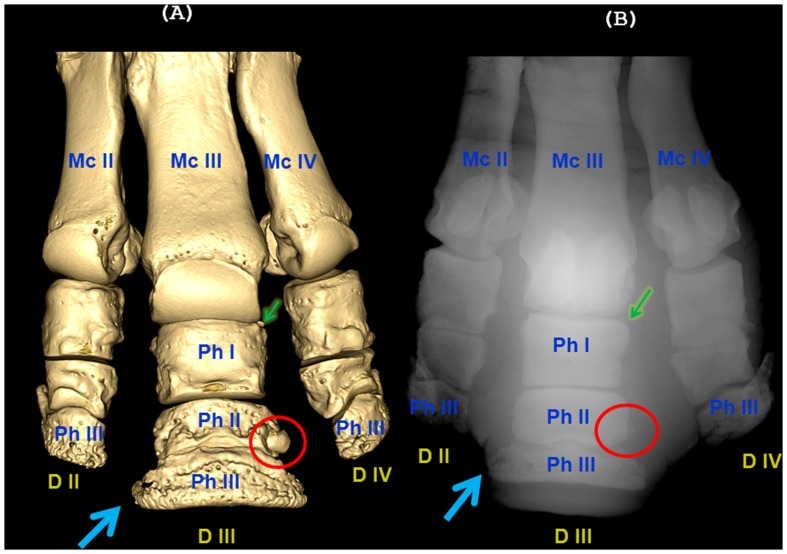
Bone pathology of the distal left front limb in rhinoceros 3. Computed tomographic tridimensional images of the distal autopodium, dorsal aspect (A) and the correspondent digital radiographic images, palmaro-dorsal view (B). The third or middle digit (D III) presents fractures on all three phalanges: the first phalanx (Ph I) - chip fracture with a small fragment (4.7 mm diameter) on the dorso-lateral aspect (A, B- small arrows); the second phalanx (Ph II) - dorso-lateral fracture with a displaced fragment (18,3×14,4×12,1 mm, A, B-circles); the third phalanx (Ph III) - chip fracture of the medial palmar process (processus palmaris medialis, A, B- large arrows).

A total of 203 autopodial bones were investigated in this study. Among them, 58 bones (28.5%) at 95 sites in both Indian and white rhinoceroses presented pathological changes (Table 1). These were comprised of a large spectrum of lesions including: cortical sclerosis, proliferative new bone formation and bone remodelling with loss of normal shape (25/95; 26.3%, Fig. 4, 5, 6), intra- and periarticular mineralized bodies or bony fragments (23/95; 24.2%, Fig. 5, 6, 7), fractures (19/95; 20.0%, Fig. 3, 4, 6), periosteal proliferation (continuous and interrupted; 15/95; 15.7%), and osteolysis and bone rarefaction (11/95; 11.7%, Fig. 4, 5, 6). Enlargement of the linear radiolucent areas along the distal border of the distal phalanx termed “vascular channels”, and changes in the trabecular pattern were also found. Bone cystic formation (n = 1) and ankylosis (n = 1, Fig. 4) were the rarest osteopathologies. Concomitant presence of several lesions was similar in appearance to end stage degenerative joint disease (DJD), osteoarthrosis and/or osteoarthritis. Of the 95 sites with bone pathologies, significantly more were situated in the front limbs than in the hind limbs (n = 59 vs. n = 36, respectively; Chi-square = 5.568, *P* = 0.023). Comparison between the medial and lateral digits revealed a higher prevalence of osteopathologies on the medial digit (Fig. 2) in the hind (n = 17 vs. n = 6; Chi-square = 5.261, *P* = 0.035) but not in the front (n = 26 vs. n = 17; Chi-square = 1.884, *P* = 0.222) limbs. The third or middle digit was less affected than the medial digit in the hind limbs (n = 17 vs. n = 5; Chi-square = 6.545, *P* = 0.017) as well as in the front limbs (n = 26 vs. n = 9; Chi-square = 8.257, *P* = 0.006). When prevalence of osteopathologies per digit was compared for both front and hind limbs combined, there were more osteopathologies in the medial digit (n = 43) when compared to the lateral digit (n = 23; Chi-square = 6.061, *P* = 0.019) or the middle digit (n = 14; Chi-square = 14.754, *P* = 0.00015). While the medial digit presented more osteopathologis when compared to the lateral digit, this was not the case when the middle, or third, digit was compared to the lateral one. No difference was found in either front or hind limbs or when both feet were combined when prevalence of osteopathologies was compared between the middle and lateral digits. The only difference found between the medial and lateral digits when osteopathologies’ prevalence was compared was in the occurrence of periosteal reaction (n = 12 vs. n = 2, respectively; Chi-square = 7.143, *P* = 0.0129). There were also more periosteal reaction (n = 12 vs. n = 1; Chi-square = 9.308, *P* = 0.00342) and bone remodelling (n = 14 vs. n = 1; Chi-square = 11.267, *P* = 0.0010) in the medial digit when compared to the middle digit. The digits (including metapodial, phalangeal and sesamoidal bones) were by far the most prevalent site for osteopathologies, presenting more osteopathologies than in all other studied bones combined (n = 66 vs. n = 29; Chi-square = 14.411, *P* = 0.00019). Of these, the phalanges (69.4% of the lesions), metapodials (9.4% of the lesions) and proximal sesamoids (5.2% of the lesions) were the most affected. Within the digits, the highest prevalence of osteopathologies (36.8% of the lesions) was in the third phalanx (n = 35, with 17 lesions in the hind legs and 18 in the front legs), more than the second phalanx (n = 17; Chi-square = 6.231, *P* = 0.018) or the first phalanx (n = 14; Chi-square = 9.000, *P* = 0.0038). There was no difference in osteopathologies prevalence between the first and second phalanges. The carpal and tarsal bones presented a wide variety of pathologies such as fractures, focal osteolysis, enthesiophytosis, osteophytosis, cortical osteogenesis, bone remodeling, and ankylosis.

Many, but not all, lesions found in CT images were also identified in the digital radiographs (Table 1 and Fig. 7).

## Discussion

### Discrepancy between the Reported Bone Pathologies in Elephants and Other Megaherbivores

The tremendous discrepancy between the reported and actual cases of bone pathology suggests the need for some new accounting. Taking into consideration the bone’s invariable response to any disease as osteolysis and osteogenesis [27] and the universality of etiologies [15], [18], there is no reasonable explanation why bone pathology is so frequently reported in captive elephants and seemingly almost non-existent in captive rhinoceroses or other megaherbivores [28].

We do not fully understand the cause for this discrepancy, but we can glean a clue from the medical records. While elephants are easier to train for handling and managing [29], for other megaherbivores diagnostic imaging involves sedation or general anesthesia, therefore requiring profound clinical justification. As a consequence, radiographic procedures became an integral part of the elephants’ medical management and only rarely used in the other megaherbivores. Radiography and CT have long proved useful diagnostic and research tools for the assessment of normal anatomy and bone diseases in elephants [16]–[18], [30]–[34]. On the other hand, bone pathology in rhinoceroses, as in other megaherbivores, has long been overlooked since radiographic techniques, protocols, normal radiographic anatomy, and pathology have not been established to date.

### Possible Origins for Distal Limb Osteopathology

The large number of bone pathologies found in the present study could carry all-important hints about their origin. They may indicate inadequate husbandry conditions [13], [14], exposure to traumatic events, overweight followed by excessive bone mechanical load, nutritional deficiencies, and/or aging alterations, but without clear correlations with other diagnostic tools, their origin remains merely speculative. An effort to gain a better understanding of these pathologies may help us recognize their potential causes and improve their treatment, providing new insights into problems such as chronic foot disease.

### Prevalence and Distribution of Bone Lesions

The predilection of lesions includes the front legs (have to sustain a higher share of the body weight), medial digits [postural deviations from the mesaxonic symmetry; 24,35], and distal phalanges (closest contact with the ground surface). These findings put together may suggest captivity-related pathologies that are induced by a combination of overweight, insufficient exercising and prolonged treading on hard surfaces. In the absence of data on the feet of rhinoceroses in their natural habitat, it would be difficult to pinpoint the collection of conditions that culminate in the observed poor *status quo* of the captive rhinoceros feet unless a controlled long-term study comparing different management techniques and enclosure design is conducted.

### Reporting a Remarkable Number with a Wide Variety of Bone Lesions in Rhinoceros’ Distal Limb

Computed tomographic images provided excellent bone details of the distal limbs depicting numerous bone pathologies, which are described for the first time in rhinoceroses. This report brings to attention the presence of osteopathology in white rhinoceroses’ foot. Even more worrying is the fact that the largest number of osteopathologies was present in one of the white rhinoceroses that showed no discernable related clinical signs. Furthermore, the current study makes known that chronic foot disease in Indian rhinoceros is not restricted only to soft tissues, but also involves the underlying bones, with severe and highly prevalent osteopathologies. The nature of these pathologies is challenging to interpret. The main causes for osteopathologies are highly diverse and universal [15], [18]. They are classified as: a) non-infectious, namely congenital, developmental, nutritional, metabolic, old age, traumatic injuries, lack of sufficient exercise, overweight, neoplasia, and soft tissue related pathologies leading to postural changes followed by conformational changes of the bones and associated joints and b) infectious, resulting in septic osteitis, osteomyelitis, arthritis, and osteoarthritis. Taking into consideration this wide etiologic spectrum, bone pathology in rhinoceroses must be analogous and present just like in any other terrestrial mammal. Indeed, the majority of bone lesions in rhinoceroses reported here are similar to the reported elephant’s foot osteopathologies [15]–[18]. Some of them, such as central tarsal bone fractures in rhinoceros 1 concealed by new bone production, are well known in small animals [27]. Other pathologies are similar with the ones found in horses [36]–[43]. Nevertheless, comprehensive knowledge of the specific anatomy in rhinoceroses’ distal foot is a prerequisite as the extrapolation from the closest related domestic perissodactyle, the horse, or between different rhinoceroses’ species, seems not always straightforward [44], [45].

### Computed Tomography - Digital Radiography Comparison

The major disadvantage of radiography is the superimposition of a 3D structure (bones) on a 2D plane, which makes it difficult and sometimes impossible to distinguish particular details [46]. Additionally, radiography of mega-vertebrates poses special technical challenges due to the massive size of the animals [32], [34]. Digital radiography was able to depict several of the encountered osteopathologies (Table 1), whereas CT images provided the most accurate imaging diagnosis. These differences are in concordance with previous published data in horses [41], [42], [47], [48] reinforcing the conclusion that CT is very useful for diagnosis of subtle bone lesions when radiography remains inconclusive [49].

However, due to the impossibility of performing distal limb CT examination on living adult megaherbivores, radiography remains the only imaging technique to be used for bone pathology diagnosis in these large, wild animals situated under field conditions. High-resolution CT and innovative, synchronized CT-DR imaging can be used as supportive, non-invasive diagnostic tools for post mortem studies, providing valuable reference data for imaging and studying normal anatomy and pathology of distal limb [50]–[53].

## Conclusions

Our preliminary evidence reported in this study indicates that distal limb’s osteopathologies in megaherbivores are much more prevalent than diagnosed. In the absence of diagnosis and identification of the conditions that lead to such bone pathologies, measures cannot be taken to prevent them and treatments cannot be initiated to alleviate suffering. It is highly recommended that radiographic examination of the distal limbs become a standard diagnostic tool in these animals, acknowledging that the first prerequisite step towards a better clinical management is to elucidate the pathology involved.

Specifically, the only five remaining species of rhinoceroses [23] are all threatened by extinction to varying degrees [54]–[59] and for some of them the only future might be exclusively in captivity. Despite their long history in captivity, extending at least to Roman times [60], even the future of some rhinoceros species in zoological collections is still uncertain [61]–[64]. Therefore, an improved knowledge of radiologic diagnosis is important for the animals’ welfare and should be used when developing the most appropriate wildlife management and conservation strategies.
